# Gender differences in self-perceived competence vs. objective knowledge of negative symptoms in Polish Early Career Psychiatrists

**DOI:** 10.1192/j.eurpsy.2026.11965

**Published:** 2026-07-31

**Authors:** A. J. Krupa, S. Galderisi, A. Mucci, Z. Sołtys, T. Gondek, J. Rausch, M. Siwek

**Affiliations:** 1 Department of Affective Disorders, Jagiellonian University Medical College, Krakow, Poland; 2 Department of Mental and Physical Health and Preventive Medicine, University of Campania “Luigi Vanvitelli”, Naples, Italy; 3 Institute of Zoology and Biomedical Research, Laboratory of Experimental Neuropathology, Jagiellonian University, Krakow; 4 Institute of Social Studies, University of Lower Silesia, Wroclaw, Poland; 5 Department of Psychiatry and Psychotherapy, Medical Center – University of Freiburg, Freiburg, Germany

## Abstract

**Introduction:**

Clinical competences and knowledge about the negative symptoms (NS) of schizophrenia (SZ) represent a vital yet unmet training need in the Polish Early Career Psychiatrists (ECPs). A recent pilot analysis reported that the majority of Polish ECPs present limited knowledge in the evaluation and management of NS. It also showed that male gender was linked to higher levels of self-reported competence in NS management, but not to the ability to identify the NS domains (Krupa et al. Front Psychiatry 2025;16:1643722).

**Objectives:**

To explore gender differences in self-reported sense of competence and actual knowledge about NS of SZ.

**Methods:**

This work is based on the Polish sample of 140 ECPs who participated in the online survey assessing competence and knowledge in evaluating and managing NS of SZ. In this post-hoc analysis, female and male ECPs were compared with regard to training characteristics, general sense of clinical competence and knowledge about NS. The variable representing general clinical competence in NS was constructed by summing responses regarding perceived competence in: 1) consulting people presenting NS vs. positive symptoms of SZ, 2) consulting people with persistent NS of SZ, 3) evaluating NS, 4) managing NS. The knowledge variable was derived from responses to questions assessing factual knowledge, based on guidance from the European Psychiatric Association, the World Federation of Societies of Biological Psychiatry, and the Canadian Network for Mood and Anxiety Treatments, as described in (Krupa et al. Front Psychiatry 2025;16:1643722). Chi-square tests were used to compare qualitative variables, ANOVA was used to compare quantitative variables among groups.

**Results:**

81 ECPs were female and 56 were male. Female vs. male groups were comparable with regard to years of experience in the mental health system (4.9±0.38 vs. 4.95±0.48, p=0.93), engagement in clinical research (24.7% vs. 38.9%, p=0.09), participation in theoretical courses on NS (43.2% vs. 49.2%, p=0.5) or placement in clinics/wards specialized in SZ care (76.5% vs. 78%, p>0.99) as part of specialist training curriculum, or extracurricular courses on NS (60.5% vs. 66.1%, p=0.6). Male ECPs displayed a higher self-reported sense of general clinical competence in NS than female ECPs (12.45±0.39 vs. 11.4±0.32, p=0.04), despite similar level of knowledge (1.71± 0.11 vs. 1.74±0.1, p=0.85).

**Conclusions:**

This work indicates a gender-based discrepancy between self-perceived competence in the domain of NS among Polish ECPs. This aligns with earlier studies showing that males tend to overestimate their competence more than females (Kilic et al. Eur Psychiatry 2023;66:e89). These findings underscore the need for training programs and clinical supervisors to support female ECPs in building their confidence, while also encouraging male ECPs to critically assess areas where further training is needed.

**Disclosure of Interest:**

None Declared

